# An impending inhibitor useful for the oil and gas production industry: Weight loss, electrochemical, surface and quantum chemical calculation

**DOI:** 10.1038/s41598-017-13877-0

**Published:** 2017-11-02

**Authors:** Ambrish Singh, K. R. Ansari, Xihua Xu, Zhipeng Sun, Ashok Kumar, Yuanhua Lin

**Affiliations:** 1 School of Materials Science and Engineering, Southwest Petroleum University, Chengdu, 610500 Sichuan China; 2 State Key Laboratory of Oil and Gas Reservoir Geology and Exploitation, Southwest Petroleum University, Chengdu, Sichuan 610500 China; 3 Department of Chemistry, Indian Institute of Technology, Banaras Hindu University, Varanasi, 221005 U.P. India; 4 Department of Chemistry and Biochemistry, Arizona State University, Tempe, Arizona 85287-1604 United States of America

## Abstract

The influence of a Schiff base namely N,N′-(pyridine-2,6-diyl)bis(1-(4-methoxyphenyl) methanimine) (PM) on the corrosion of J55 and N80 steel in 3.5 wt.% NaCl solution saturated with CO_2_ was evaluated using weight loss, potentiodynamic polarization, electrochemical impedance spectroscopy (EIS), X-ray diffraction (XRD), contact angle, scanning electron microscopy (SEM), atomic force microscopy (AFM) and scanning electrochemical microscopy (SECM). Potentiodynamic polarization results suggested that the inhibitor acted as a mixed type inhibitor by reducing both anodic and cathodic reactions. The adsorption of PM on the J55 and N80 steel surface obeyed the Langmuir adsorption isotherm. XRD, contact angle, SEM, AFM and SECM studies revealed that the surface of the metal was quite unaffected after the addition of inhibitor. Quantum chemical calculations and molecular dynamic simulation support the experimental results well.

## Introduction

The varying amount of gases and the high concentration of salts in water are the major constituents of the oil and gas production industry^1^. Of the gases, carbon dioxide in the presence of high chloride concentrations is the most common corrosive medium in the petroleum industry, and such corrosion is said to be sweet corrosion^2–4^. Infrastructure such as pipe lines and oil well processing equipment in the oil and gas industry is made of carbon steel due to its lower cost. Although the carbon steel is resistant towards corrosion, in presence of a high content of chloride aqueous solutions of carbon dioxide, a significant corrosion problem arises. This corrosion problem results in a tremendous loss of the revenue to the oil and gas industry, either in the form of loss in production or repair costs for the production unit. Additionally, an indirect impact of corrosion occurs over the environment and ecology^5^.

The problems arising from carbon dioxide corrosion have lead to the development of various methods of corrosion control. Of these methods, injection of corrosion inhibitors has proven to be most practical and economic method due to its simplicity of use^6^. Many organic compounds have been tested as corrosion inhibitors, but heteroatoms containing nitrogen, oxygen, and sulphur are the most commonly used inhibitors because the heteroatoms can easily interact with the metal surface by donating their lone electron pair. Hence, most of the organic compounds containing heteroatoms and multiple bonds act as good corrosion inhibitors^7–9^, and Schiff bases are the best known examples in this category. The review of the literature reveals that despite the superlative inhibition characteristics of Schiff bases in general, this class of compound has so far not been exploited as a corrosion inhibitor for carbon dioxide^10,11^. By keeping our eyes on the losses due to corrosion and environmental safety, we have synthesized N,N′-(pyridine-2,6-diyl)bis(1-(4-methoxyphenyl)-methanimine), which shows various types of biological activity such as antibacterial, antimicrobial, antitubercular, local anaesthetic, anti-inflammatory, anti-convulsant, anti-viral and anti-cancer^12^.

In the present study, N,N′-(pyridine-2,6-diyl)bis(1-(4-methoxyphenyl)-methanimine) has been synthesized, and its corrosion inhibition effect was tested on both J55 and N80 steels in 3.5% NaCl solution saturated with carbon dioxide using gravimetric methods, potentiodynamic polarization, electrochemical impedance spectroscopy (EIS), X-ray diffraction (XRD), UV-visible spectroscopy, contact angle measurement, scanning electron microscopy (SEM), atomic Force Microscopy (AFM), scanning electrochemical microscopy (SECM), quantum chemical calculations and molecular dynamic simulation (MD).

## Experimental procedures

### Inhibitor synthesis

**Figure 1:**
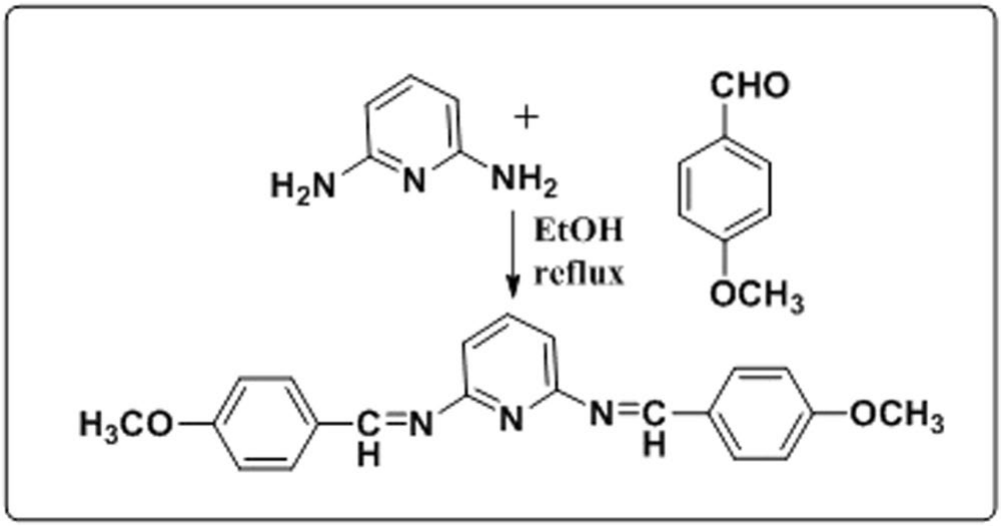
Synthetic scheme of inhibitors.

2,6-Diaminopyridine (0.1 mol) and 4-methoxybenzaldehyde (0.2 mol) were refluxed in ethanol (20 mL) for approximately 5 h. The solid mass thus obtained was filtered and further recrystallized from ethanol^12^. The synthesis scheme is shown in Fig. 1, and the ^1^H-NMR, IR spectrum is given in supplementary file S1 and S2 respectively.

The detailed characterization is as follows:

#### N,N′-(Pyridine-2,6-diyl) bis(1-(4-methoxyphenyl)-methanimine) (PM)

^**1**^ **H-NMR** (500 MHz, DMSO-d_6_) δ (ppm): 3.827(OCH_3_), 6.819–7.062 (CH Pyridine) 7.802–7.819 (CH benzene), 9.123 (=CH).

**IR** (KBr cm^−1^): 3053 (Ar-CH), 2875 (C-H aliphatic), 1715 (C = O), 1617 (C = N), 1580 (C = C).

### N80 and J55 steel specimens

The composition of the steel samples is as follows: J55 steel (wt%): C 0.24; Si 0.22; Mn 1.1; P 0.103; S 0.004; Cr 0.5; Ni 0.28; Mo 0.021; Cu 0.019; Fe remainder^13^ and N80 steel of (wt%): C 0.31; Si 0.19; Mn 0.92; P 0.010; S 0.008; Cr 0.2; Fe remainder. The steel coupons are flat with a dimension of 5.0 cm × 2.5 cm × 0.2 cm and 2.0 cm × 1.0 cm × 0.025 cm used for gravimetric and electrochemical experiments, respectively. Only one end face (1.0 cm^2^) was exposed, and the rest was sealed by epoxy resin. All the steel coupons were abraded through 600, 800 and 1200 grit silicon carbide metallurgical paper, degreased in acetone, washed with anhydrous ethanol, and then dried at room temperature and finally kept in the desiccators^14^.

### Experimental solution

In the present study, the test solution is 3.5% NaCl saturated with carbon dioxide that was prepared by passing carbon dioxide gas through the solution for 120 min at a pressure of 6 MPa until the pH of the solution became 4 ± 0.05 and, when necessary, the pH was adjusted with small amounts of NaHCO3 or HCl. The 3.5% NaCl solution was continuously saturated with CO_2_ throughout the experiment, and nitrogen gas was passed through the solution to minimize the oxygen concentration prior to each test. All the experiments were performed in static, unstirred solutions.

### Weight loss experiments

The gravimetric experiments were done by immersing the steel samples (J55 and N80) in 3.5% NaCl solution saturated with CO_2_ for 7 days. The corrosion rate (*C*_R_) and inhibition efficiency (*η*%) were calculated using the following equations:

1 $${\rm{C}}{}_{{\rm{R}}}(\mathrm{mm}/{\rm{y}})=\frac{{\rm{87}}{\rm{.6W}}}{{\rm{atD}}}$$

2 $$\eta  \% =\frac{{C}_{{\rm{R}}}-{C}_{{\rm{R}}({\rm{i}})}}{{C}_{{\rm{R}}}}\times 100$$

where *W* is the weight loss of the specimen (mg), *a* is the area of the specimen (cm^2^), *t* represents the immersion time (*h*), and *C*_R_ and *C*_R(i)_ are the corrosion rates in the absence and presence of the inhibitor molecules, respectively.

### Electrochemical methods

All electrochemical studies, i.e., potentiodynamic polarization and electrochemical impedance spectroscopy (EIS) experiments were performed using a standard three electrode cell, which consists of J55 and N80 steel strips as working electrodes, a graphite rod as the counter electrode and Ag/AgCl as the reference electrode. The stable value for the open circuit potential was achieved by immersing the working electrodes in the test solution for 30 min. Potentiodynamic polarization and EIS measurements were performed using the Autolab Potentiostat/Galvanostat electrochemical analysis device. EIS measurements were carried out in the frequency range of 100 kHz to 0.00001 kHz at the amplitude of 10 mV, peak to peak.

Potentiodynamic polarization was carried out by changing the potential from-250 mV to +250 mV vs open circuit potential (OCP) at a constant sweep rate of 1 mV/s.

### Surface analysis (SEM, AFM and XRD)

Surface analysis of steel samples in the absence and presence of inhibitor was performed using the TESCAN VEGA II XMH instrument and AFM studies were performed using the NT-MDT SOLVER Next AFM/STM instrument. The scanned size of each sample used in AFM is 10 μm × 10 μm.

The films formed on the surface of the steel specimens were analysed using an X-ray diffractometer, X Pert PRO incorporated with High Score software.

### Contact angle and SECM measurements

Contact angle measurements were performed using the sessile drop technique with the help of the DSA100 Kruss instrument made in Germany. SECM studies were carried out using an electrochemical work station of CHI900C model consisting of a three-electrode assembly.

### Computational methods

Density Functional Theory (DFT) calculations are an important tool to predict the reactivity or stability of inhibitor molecules and were performed using the Gaussian 09 program^15^. Gauss View 5.0.8 was used to prepare the input files of inhibitor molecules^16^. The optimization of the inhibitor molecules was done using a 6–31 G (d, p) basis set. All the calculations have been carried for the aqueous phase, both for neutral and protonated inhibitor molecules. Quantum chemical parameters such as energy of the highest occupied molecular orbital (*E*_HOMO_), energy of the lowest unoccupied molecular orbital (*E*_LUMO_), electronegativity (*χ*), hardness (*η*), softness (*σ*) and the fraction of electrons transferred (Δ*N*) were calculated and discussed.

The ionization potential (*IP*) and electron affinity (*EA*) energies are correlated with the HOMO and LUMO of the inhibitor molecules, respectively, and can be expressed as follows^17–20^:

3 $$IP=-{E}_{HOMO}$$

4 $$EA=-{E}_{LUMO}$$

Additionally, electronegativity (*χ*), global hardness (*η*) and global softness (σ) are given as follows^21^.

5 $$\chi =-\frac{1}{2}({E}_{HOMO}+{E}_{LUMO})$$

6 $$\eta =-\frac{1}{2}({E}_{HOMO}-{E}_{LUMO})$$

7 $$\sigma =\frac{1}{\eta }$$

The fraction of electrons transferred (Δ*N*) from the inhibitor molecules to the metal surface was calculated using the values of *χ* (electronegativity) and *ƞ* (global hardness) and can be expressed as follows^22^.

8 $${\rm{\Delta }}N=\frac{\varphi -{\chi }_{{\rm{inh}}}}{2({\eta }_{{\rm{Fe}}}+{\eta }_{{\rm{inh}}})}$$

where *ϕ* is the work function and χ_inh_ is the electronegativity of inhibitor molecule, *ƞ*_Fe_ and *ƞ*_inh_ denote the absolute hardness of iron and the inhibitor molecule. The values of *ϕ* and *ƞ*_Fe_ are taken as 4.82 and 0 eV mol^−1^ ^23^.

### Molecular dynamics simulation

The interaction between the inhibitor molecules and the metal surface was studied using molecular dynamics (MD) simulations using the Forcite module of the Materials Studio 6.0 program developed by Accelrys, Inc.^24,25^. In this method, the most densely packed and stable iron surface was chosen, i.e., Fe-(110) for the adsorption study^26^. The MD simulation was performed at the temperature of 313 K, controlled by the Andersen thermostat and NVT ensemble, with a time step of 1.0 fs and simulation time of 10 0 0 ps, using the COMPASS^27^ force field.

### Fukui functions

The calculation of the Fukui functions was performed using UCA-FUKUI v 1.0 software^28^ using the Finite Difference (FD) method with the help of the output file from Gaussian 09. The Fukui function (*f*_k_) is the first derivative of the electronic density $\rho (\vec{r})$ with respect to the number of electrons N, in a constant external potential ${\mathscr{V}}(\vec{r})$ and is written as follows^29^.

9 $${f}_{k}={(\frac{\partial \rho (\vec{r})}{\partial N})}_{{\mathscr{V}}(\vec{r})}$$

Nucleophilic and electrophilic attacks were calculated using the Finite Difference approximations method^29^:

10 $${f}_{k}^{+}={q}_{k}(N+1)-{q}_{k}(N)\quad \quad ({\rm{for}}\,{\rm{nucleophilic}}\,{\rm{attack}})$$

11 $${f}_{k}^{-}={q}_{k}(N)-{q}_{k}(N-1)\quad \quad ({\rm{for}}\,{\rm{electrophilic}}\,{\rm{attack}})$$

Here, *q*_k_ represents the gross charge of the atom. The charges on the anionic, neutral and cationic species are denoted by *q*_k_ (*N* + 1), *q*_k_ (*N*) and *q*_k_ (*N* − 1) respectively.

## Results and Discussion

### Weight loss

#### Consequence of concentration

**Figure 2:**
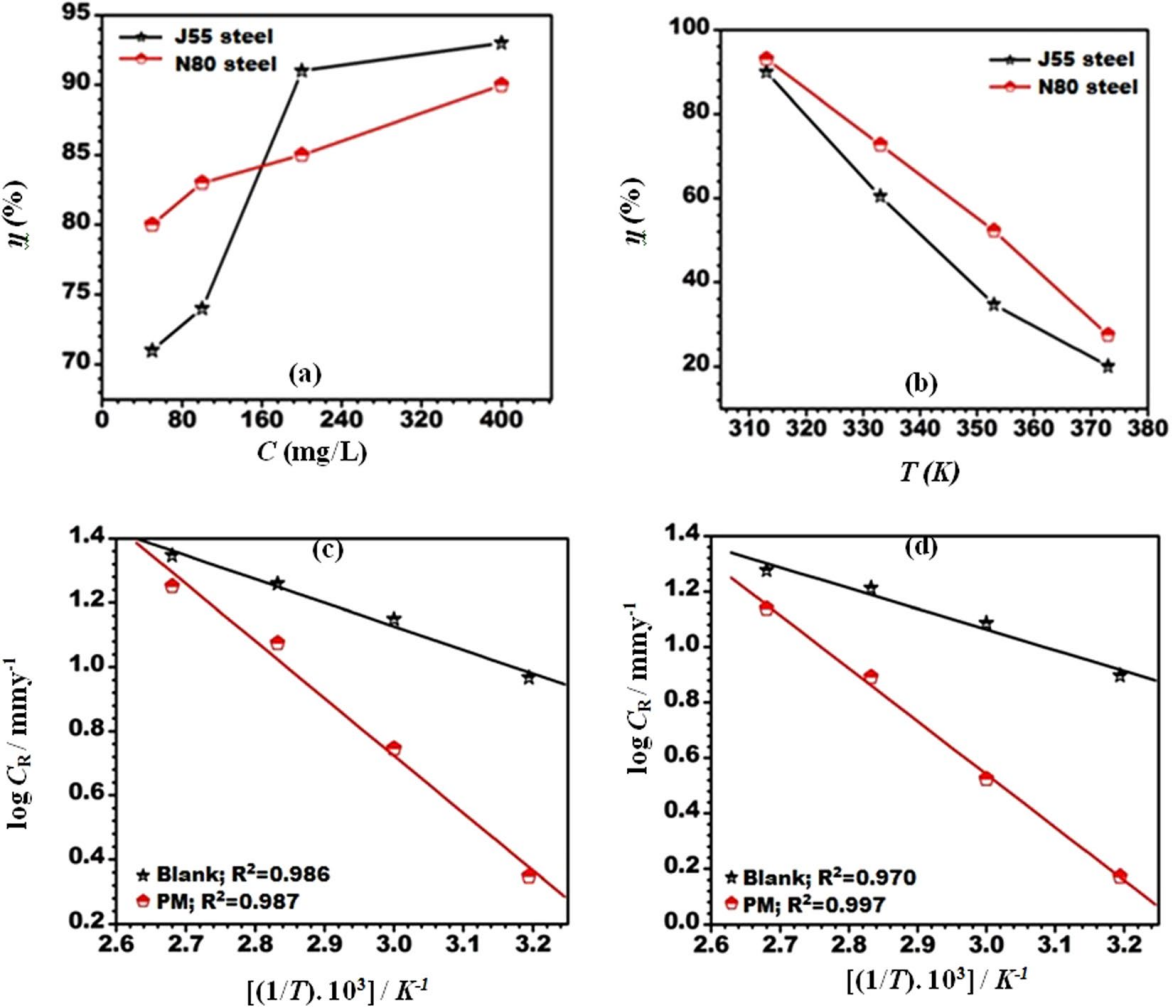
(**a**) Variation of inhibition efficiency (*η* %) with inhibitor concentration at 313 K. (**b**) Variation of inhibition efficiency (*η* %) with temperature. (**c**,**d**) Arrhenius plots of the corrosion rate (*C*_R_) of (**c**) J55 steel (**d**) N80 steel in the absence and the presence of an optimum concentration of inhibitor.

The percentage inhibition efficiency with the increase in inhibitor concentration is shown in Fig. 2a, which shows that the inhibition efficiency increases as the inhibitor concentration increases, suggesting that a greater number of inhibitor molecules is adsorbing over the active sites of the metal and thus preventing the direct contact between the metal and the aggressive solution. The highest inhibition efficiency obtained at 400 mg/L is 93% (J55 steel) and 90% (N80 steel). However, a further increase in the inhibitor concentration provides no significant change in the value of the inhibition efficiency. Therefore, 400 mg/L has been selected to be the optimum concentration.

#### Consequence of temperature

The variation in the inhibition efficiency with increase in the temperature from 313 to 373 K at the optimum inhibitor concentration is shown in Fig. 2b. Figure 2b shows that the inhibition efficiency decreased with the increase in the temperature for both J55 and N80 steels due to desorption of the inhibitor molecules from the metal surfaces^30^.

The activation energy for the corrosion process was calculated using the Arrhenius equation:

12 $$\mathrm{log}\,{C}_{R}=\frac{-{E}_{a}}{2.303RT}+\,\mathrm{log}\,\lambda $$

where *E*_a_ represents the activation energy, *R* is the universal gas constant, and λ denotes the pre-exponential factor. The value of the activation energy in the absence and presence of the inhibitor was calculated by taking the linear regression between log *C*_R_ and 1/T (Fig. 2c,d). The activation energy for both inhibited systems is higher than the activation energy for the uninhibited system, i.e., 14.46 kJ/mol (uninhibited J55 steel), 14.07 kJ/mol (uninhibited N80 steel). However, in the presence of inhibitor, *E*_a_ increased to 36.59 kJ/mol and 22.57 kJ/mol for J55 and N80 steel, respectively.

The high values of *E*_a_ suggest that a high energy barrier has formed in presence of inhibitor for corrosion reactions. Thus, charging or mass transfer from the metal surface is avoided due to the adsorbed inhibitor molecules.

### Electrochemical measurements

#### Electrochemical impedance spectroscopy (EIS)

**Figure 3:**
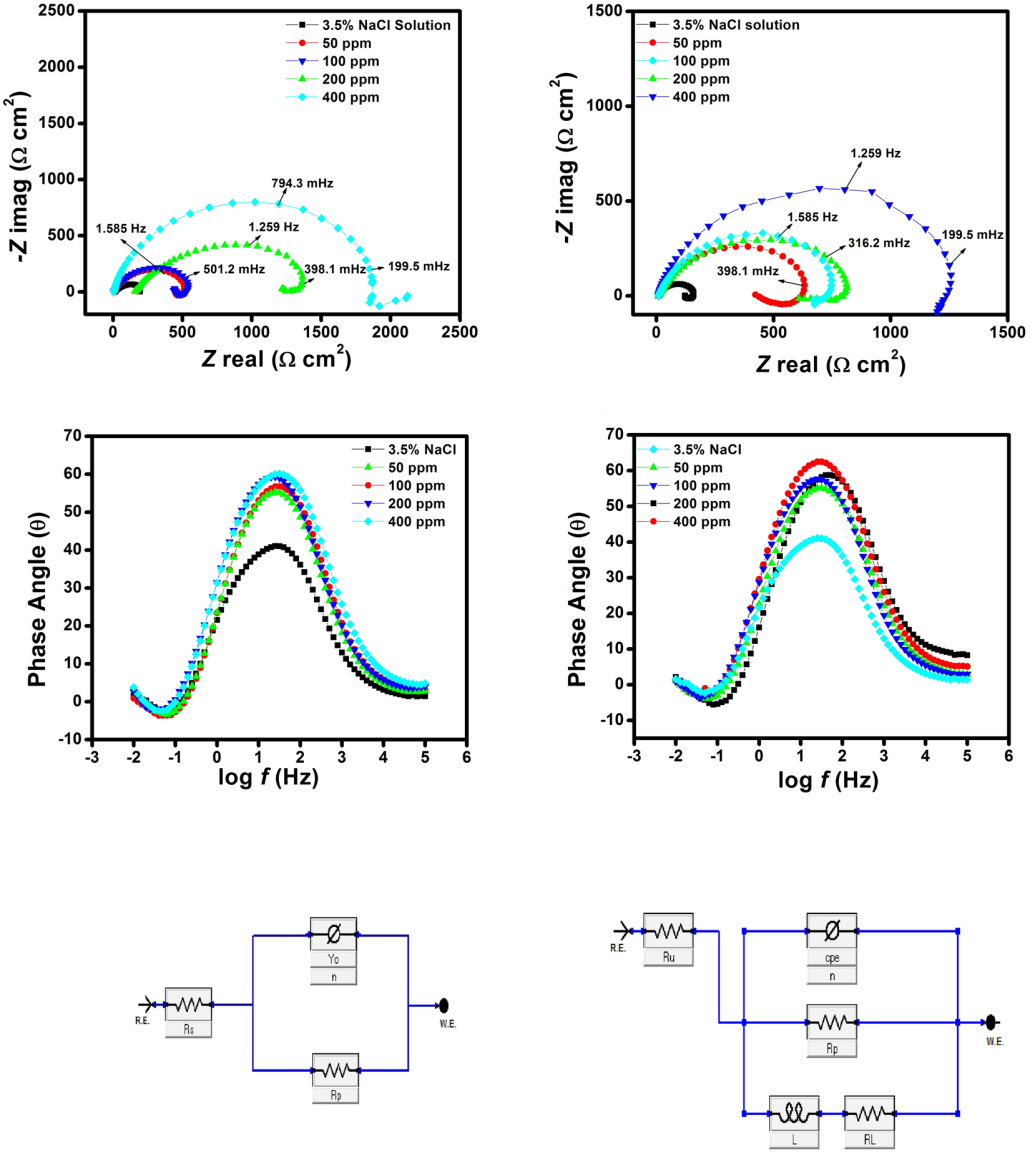
(**a**,**b**) Nyquist plots for (**a**) J55 steel (**b**) N80 steel in 3.5% NaCl saturated with CO_2_ in absence and presence of different concentration of inhibitors at 313 K. (**c**,**d**) Phase angle (log *f* vs. *α*°) plots of impedance spectra for (**c**) J55 steel (**d**) N80 steel in 3.5% NaCl saturated with CO_2_ in the absence and the presence of different concentrations of the inhibitors at 313 K. (**e**,**f)** Equivalent circuit model used to fit the EIS data.

Impedance spectra for J55 and N80 steel in 3.5% NaCl solution saturated with CO_2_ in the absence and presence of different concentrations of PM are shown in Fig. 3a–d in the form of Nyquist plots and Bode phase angle plots^31^. The Nyquist plots consist of depressed semicircles with one capacitive loop in the high frequency (HF) zone and one inductive loop in the lower frequency (LF) zone. The occurrence of an inductive loop is due to the relaxation process of *H*_ads_ or FeOH_ads_^32^. The diameter of the semicircle is increased with an increase in the inhibitor concentration, due to the adsorption of inhibitors forming a protective inhibitor film over the metal surface (Fig. 3a,b). The calculated EIS parameters from the Nyquist plots are given in Table 1.

**Table 1:** Electrochemical impedance parameters for J55 and N80 steel in 3.5% NaCl saturated with CO_2_ in the absence and the presence of different concentrations of inhibitor at 313 K.

*C*_inh_	*R*_s_	*R*_p_	*n*	*Y*_0_	*L*	*R*_L_	*η*	χ^2^
(mgL^−1^)	(Ω)	(Ω cm^2^)		(μF/cm^2^)	(H cm^2^)	(Ω cm^2^)	(%)	
**J55 steel**								
Blank	9.1	132	0.783	303	—	—	—	0.78 × 10^−2^
50	7.9	453	0.789	262	164	74	69	1.3 × 10^−2^
100	8.6	518	0.798	170	210	75	73	1.8 × 10^−2^
200	7.1	1421	0.833	95	285	92	90	1.2 × 10^−2^
400	8.1	1848	0.834	77	—	—	92	0.87 × 10^−2^
**N80 steel**								
Blank	9.6	125	0.749	299	80	33	—	0.71 × 10^−2^
50	7.1	690	0.787	208	289	135	80	0.69 × 10^−2^
100	7.7	810	0.791	150	349	184	83	1.4 × 10^−2^
200	9.1	852	0.795	85	591	304	84	1.6 × 10^−2^
400	7.5	1301	0.801	67	—	—	90	0.54 × 10^−2^

The impedance results of the EIS spectra were calculated by fitting the two equivalent circuits (Fig. 3e,f), which consist of *R*_s_ (solution resistance), *R*_*p*_ (polarization resistance), CPE (constant phase element) and *R*_L_ (inductive resistance) and *L* (inductance)^33^. The presence of *L* in the impedance spectra in the presence of the inhibitors that were investigated indicated that iron was still dissolved by the direct charge transfer at the inhibitor adsorbed electrode surface^34^. The impedance of the constant phase element is given by the following equation:

13 $${Z}_{CPE}={{Y}_{0}}^{-1}{(j\omega )}^{-n}$$

where *Y*_o_ is the magnitude of CPE, *j* is the square root of −1, and *n* is the phase shift, which can be used as a gauge of the heterogeneity or roughness of the surface, and ω is the angular frequency^35^.

In EIS, degree of difficulty in corrosion reaction is reflected by *R*_p_ values, higher the value of *R*_p_ lower is the corrosion rate. Inspection of EIS data in Table 1 shows that *R*_p_ value increases with increasing the concentration of inhibitor. This reflects that the inhibitor prevents corrosion effectively and a protective layer on the electrode surface is formed. This layer acts as a barrier towards mass and charge transfer. The precision of the fitted data was evaluated by chi-squared (*χ* ^2^). The values of *χ* ^2^ are very small (Table 1), which supports that the equivalent circuit is ideal for fitting.

The inhibition efficiencies value can be calculated according to the following equation:

14 $$:\eta  \% =(1-\frac{{R}_{p}}{{R}_{p(i)}})\times 100$$

where *R*_p_ [sum of *R*_ct_ (charge transfer resistance) and *R*_film_ (film resistance)] and *R*_p(i),_ respectively, represent the polarization resistance in the absence and the presence of different concentration of inhibitors. From the Table, we observe that the value of polarization resistance with the addition of inhibitors is increased, due to the formation of a protective film at the metal solution interface^36^. The decrease in the magnitude of CPE in presence of inhibitors (Table 1) indicates the increase in the thickness of the double layer. Additionally, the values of “*n*” in presence of inhibitors increased from 0.787 to 0.834 compared to the blank 0.783 (J55 steel) and 0.749 (N80 steel), revealing that the metal surface becomes more homogeneous in the presence of inhibitor molecules^37^.

In the Bode phase angle plots (Fig. 3c,d), at the intermediate frequency the phase angle values obtained are in the range of 38.9° to −59.8° for J55 steel and 39.1° to −61.2° for N80 steel. However, an ideal capacitor phase angle at an intermediate frequency is −90°^38^. Thus, the approach of the phase angle to −90° with the addition of inhibitors suggests that the electrochemical behaviour of corrosion becomes more capacitive^39^.

#### Potentiodynamic polarization

**Figure 4:**
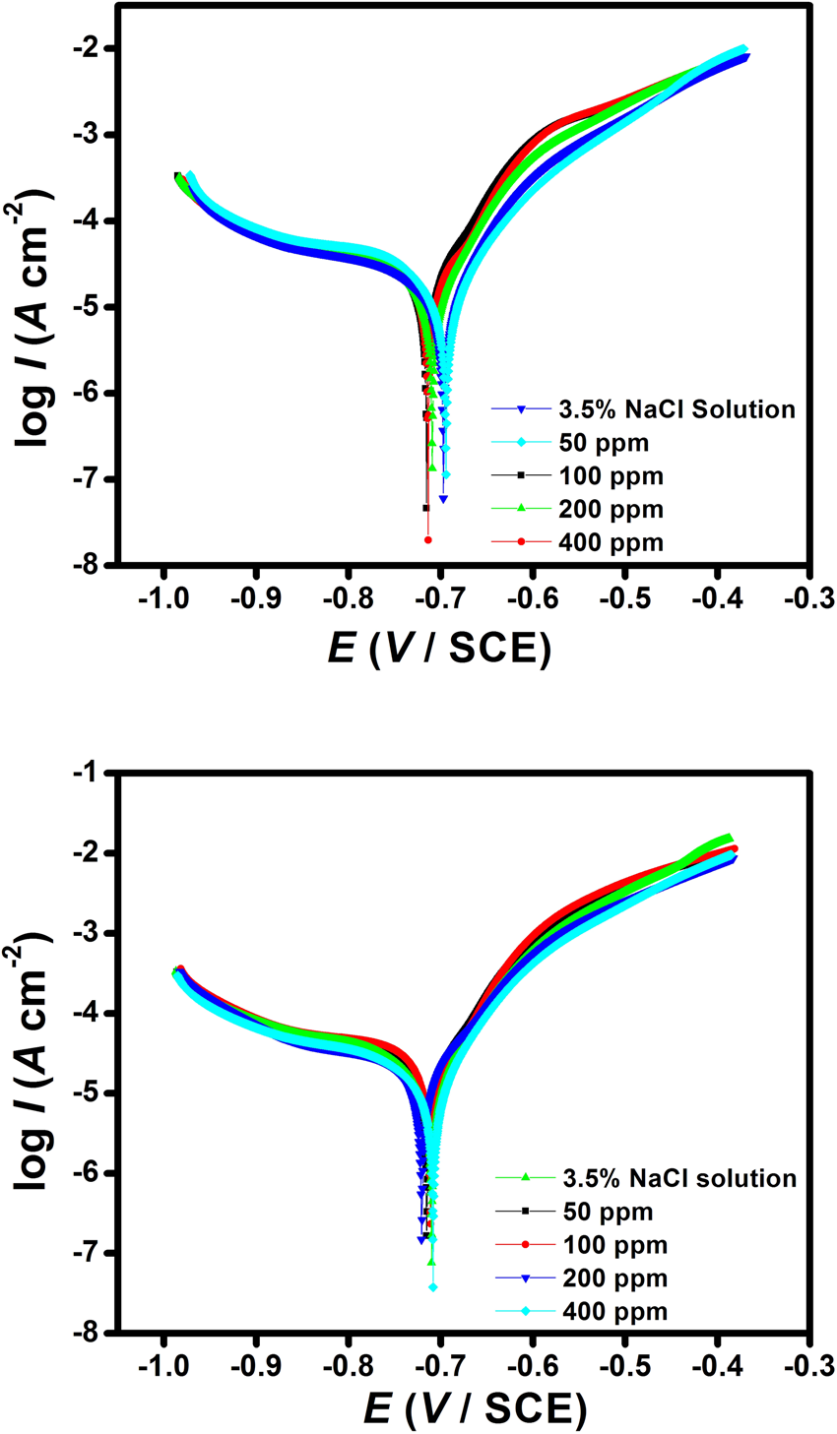
Potentiodynamic polarization curves for (**a**) J55 steel and (**b**) N80 steel in 3.5% NaCl saturated with CO_2_ in the absence and the presence of different concentration of inhibitors at 313 K.

Potentiodynamic polarization curves for J55 and N80 steel in the absence and the presence of inhibitor in 3.5% NaCl solution saturated with CO_2_ at 3131 K temperature are shown in Fig. 4a,b. The linear portion of the cathodic and anodic Tafel line allows the calculation of some valuable potentiodynamic parameters such as corrosion current density (*i*_corr_), corrosion potential (*E*_corr_), cathodic and anodic Tafel slopes (*b*_c_, *b*_a_) and inhibition efficiency (*η*%). These parameters are tabulated in Table 2 ^40^. The inhibition efficiency was calculated using the following equation:

15 $$\eta  \% =(1-\frac{{i}_{\mathrm{corr}({\rm{i}})}}{{i}_{{\rm{corr}}}})\times 100$$

where *i*_corr_ and *i*_corr(i)_ are the corrosion current density in the absence and the presence of inhibitor, respectively. Table 2 shows that as the inhibitor concentration is increased, there is a significant reduction in the corrosion current densities occurring from 94.4 µA cm^−2^ to 9.1 µA cm^−2^ for J55 steel and from 106.3 µA cm^−2^ to 10.2 µA cm^−2^ for J55 steel, which reflects that the corrosion reactions are inhibited. The shifts in the *E*_corr_ values show an almost constant trend, with a maximum change of 53 mV. Such types of *E*_corr_ value change have been attributed to a mixed type of inhibitor action^41,42^. The anodic and cathodic Tafel slope values in the presence of inhibitor for both steels (J55 and N80) shows some variations (Table 2) compared to the values in the absence of inhibitor, suggesting that in the presence of inhibitor, both the anodic and cathodic corrosion reactions are affected. Additionally, with the increase in the inhibitor concentration the values of inhibition efficiency increase, due to the formation of an adsorbed film of inhibitor molecules over the metal surface.

**Table 2:** Potentiodynamic polarization parameters for J55 and N80 steel in 3.5% NaCl saturated with CO_2_ in the absence and the presence of different concentration inhibitor at 313 K.

*C*_inh_	*E*_corr_	*i*_corr_	*β*_a_	−*β*_c_	*η*
(mgL^−1^)	(mV/SCE)	(μA/cm^2^)	(mV/dec)	(mV/dec)	(%)
**J55 steel**					
Blank	−698	94.4	154	690	—
50	−697	57.1	118	436	40
100	−721	36.1	129	636	62
200	−709	15.3	113	758	84
400	−714	8.1	85	569	91
**N80 steel**					
Blank	−715	106.3	129	179	—
50	−711	50.4	111	89	53
100	−722	34.1	97	113	68
200	−711	21.0	116	178	80
400	−724	10.2	114	102	90

### Adsorption isotherm

**Figure 5:**
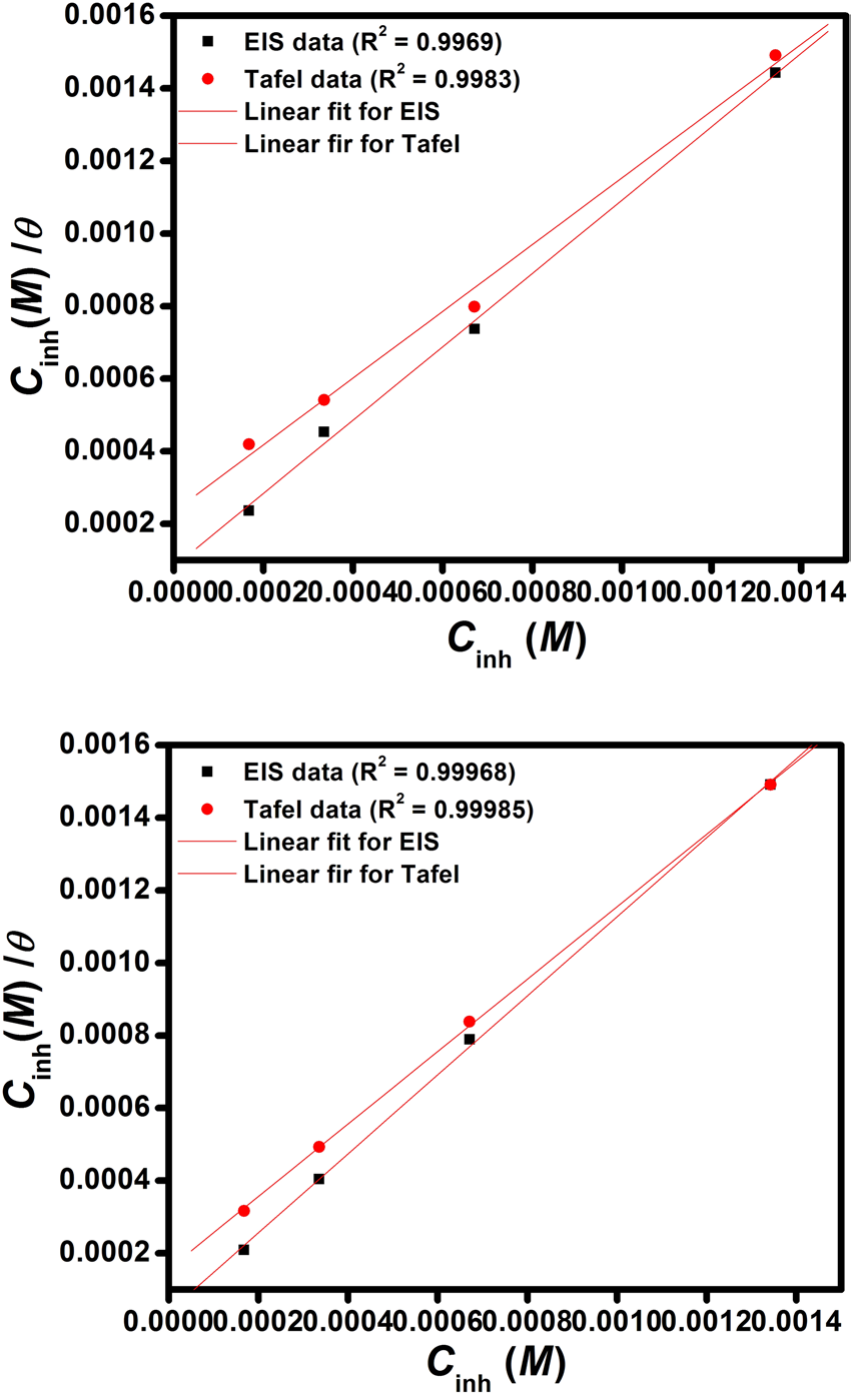
Langmuir adsorption isotherm plots for inhibitors (**a**) J55 steel and (**b**) N80 steel.

In the current investigation various isotherms were tried such as Temkin, Frumkin and Langmuir. However, the Langmuir isotherm was the best fit. The Langmuir isotherm is expressed by the following equation^43^:

16 $$\frac{{C}_{inh}}{\theta }=\frac{1}{{K}_{ads}}+{C}_{inh}$$

where *K*_ads_ is the equilibrium adsorption constant, *C*_inh_ is the inhibitor concentration and *θ* is the fraction of the surface covered by inhibitor molecules. After plotting as a graph between *C*_inh_/*θ* versus *C*_inh_, a straight line was obtained (Fig. 5a,b), with a correlation coefficient (R^2^) for J55 steel ranging from 0.9969 for EIS and 0.9983 for Tafel polarization and N80 steel from 0.99968 for EIS and 0.99986 for Tafel polarization. Values of *K*_ads_ represent the strength between adsorbate and adsorbent, i.e., larger values of *K*_ads_ imply stronger adsorption and hence, better inhibition efficiency^44–46^. The equilibrium adsorption constant (*K*_ads_) is related to the standard free energy of adsorption (Δ*G*°_ads_) through the following equation:

17 $${\rm{\Delta }}G{^\circ }_{ads}=-RT\,\mathrm{ln}(55.5{K}_{ads})$$

where, *R* is the gas constant, and *T* is the absolute temperature. The value of 55.5 is the concentration of water in the solution in mol L^−1^. The values of *K*_ads_ and Δ*G*°_ads_ are given in Table 3. The negative values of Δ*G*°_ads_ ensure the spontaneity of the adsorption process and the stability of the adsorbed film on the steel surface^47^. Generally, values of Δ*G*°_ads_ ≤ −20 kJ mol^−1^ signify physisorption, and values more negative than −40 kJ mol^−1^ signify chemisorption. The calculated value of Δ*G*°_ads_ for J55 steel with inhibitor is in the range of –37.29 kJ/mol to −33.64 kJ/ mol and for N80 steel with inhibitor is in the range of −35.32 kJ/mol to −32.60 kJ/mol (Table 3), which probably indicate that both physical and chemical adsorption would occur.

**Table 3:** Thermodynamic parameters for the adsorption of inhibitor on J55 and N80 steel in 3.5% NaCl saturated with CO_2_ in the absence and the presence of the optimum concentration of inhibitor.

Inhibitor	EIS		Tafel	
	*K*_ads_	−∆*G*°_ads_	*K*_ads_	−∆*G*°_ads_
	(10^4^ M^−1^)	(kJ mol^−1^)	(10^4^ M^−1^)	(kJ mol^−1^)
Blank	—	—	—	—
PM + J55	13.01	37.29	0.75	33.64
PM + N80	1.41	35.32	0.49	32.60

### X-Ray Diffraction (XRD)

**Figure 6:**
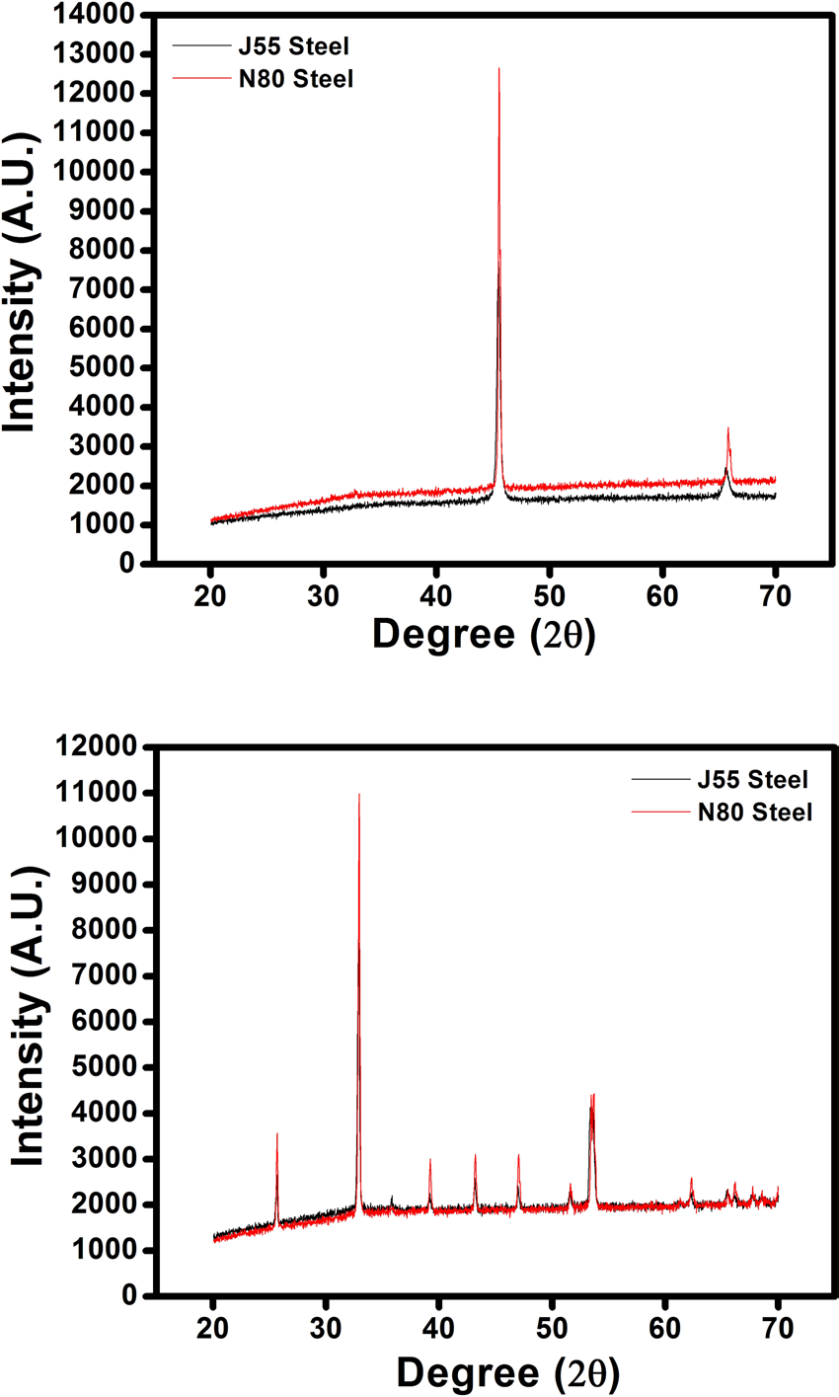
XRD spectra of (**a**) J55 sttel+ N80 steel in Inhibited solution and (**b**) J55 steel + N80 steel in 3.5% NaCl solution.

The corrosion product over the surface of the carbon steel samples was determined by X-ray diffraction, and the results are shown in Fig. 6a,b. Peaks at 2θ = 33°, 40°, 44°, 48°, 51°, 52°, and 66° can be assigned to the oxides of iron. The XRD patterns of the inhibited surface (Fig. 6b) show the presence of iron peaks only. The peaks due to oxides of iron are found to be absent^48^, attributed to the formation of a protective film of inhibitor over the metal surface.

### Contact Angle

**Figure 7:**
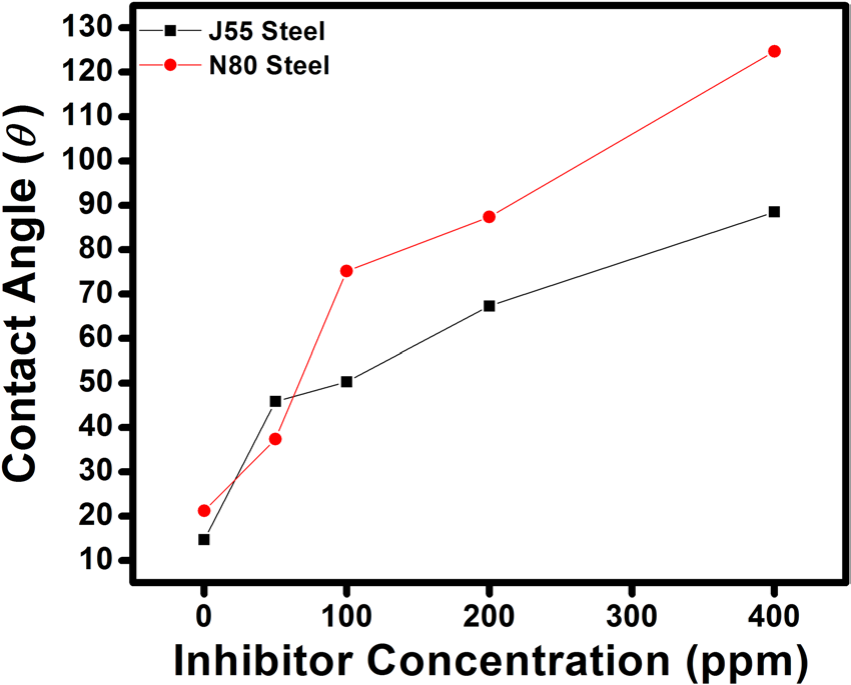
Contact angle versus inhibitor concentration plots for inhibitors.

The contact angle measurement was carried out in the absence and presence of inhibitor both for J55 and N80 steel and is shown in Fig. 7. The contact angle in the absence of inhibitor for J55 steel was measured as 21.2° and for N80 steel was 14.7°. In the absence of inhibitor, the value of the contact angle is lower, suggesting that the metal surface shows hydrophilic properties and favours water molecules to adsorb and cause more corrosion^13^. However, as the inhibitor is added, the contact angle values increased to 124.7° for J55 steel and 88.5° for N80 steel, supporting that metal surfaces became hydrophobic and repelled water molecules, and thus the corrosion process is reduced. This result confirms that the inhibitor molecules are adsorbed and make a film over the metal surface^49^.

### Surface analysis

#### Scanning Electron Microscopy (SEM)

**Figure 8:**
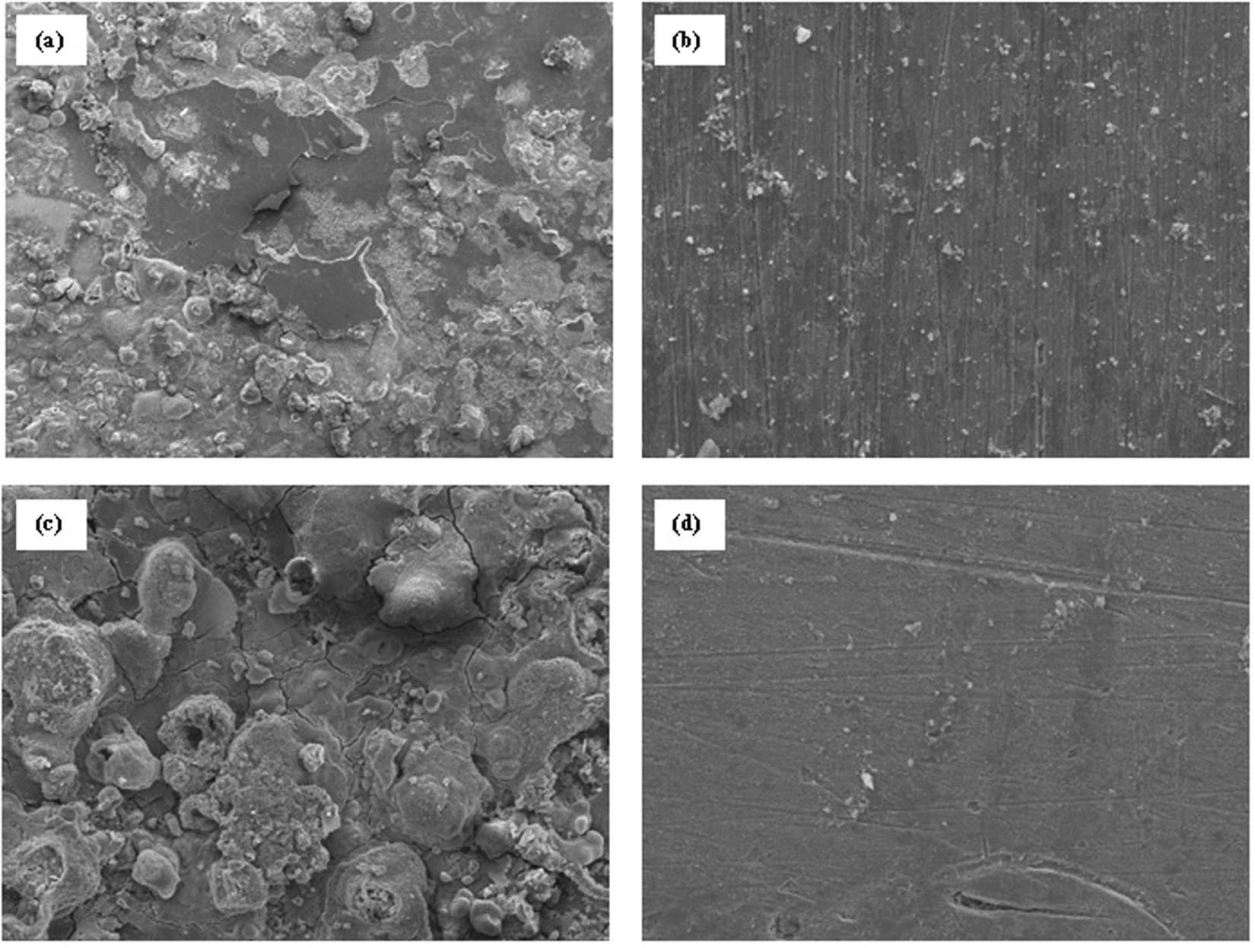
SEM images for (**a**) Blank J55 steel (**b**) PM+ J55 steel (**c**) Blank N80 steel (**d**) PM + N80 steel.

The SEM micrograph of J55 and N80 steels in the absence and presence of the optimum concentration of the inhibitor is shown in Fig. 8a–d. In the absence of inhibitor, the steel surfaces are rough due to the damage caused by the corrosive attack of the carbonic acid (Fig. 8a,b). However, in the presence of the inhibitor, the steel surfaces become smooth^50^ (Fig. 8c,d). This result further supports the presence of an adsorbed inhibitor film over the metal surface^51^.

#### Scanning Electrochemical Microscopy (SECM)

**Figure 9:**
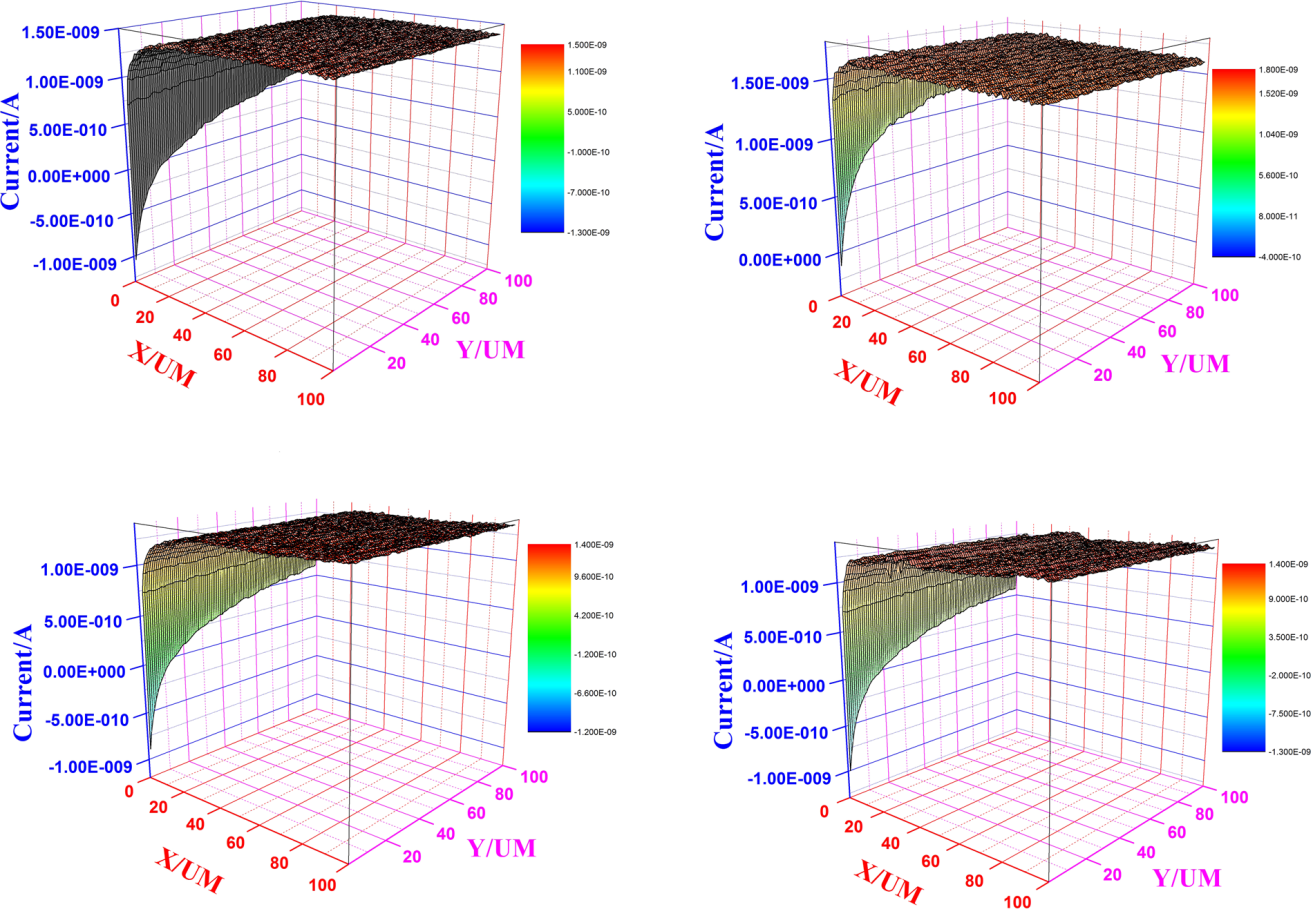
SECM figures for (**a**) Blank *y-*axis 3D-J55 steel (**b**) Blank *y*-axis 3D-N80 steel (**c**) PM + J55 steel *y-*axis 3D (**c**) PM + N80 steel *y*-axis 3D.

Figure 9a–d shows the 3-D form of SECM images of J55 and N80 steel samples immersed in 3.5% NaCl solution saturated with CO_2_^52–55^. In absence of inhibitor, when the tip of the probe was brought near to the metal surface, the current started to increase, suggesting the conductive nature of the metal surface (Fig. 9a,c)^56^. However, in the presence of the inhibitor when the probe is brought near to the metal surface, the value of the current decreases (Fig. 9b,d), suggesting that the metal surface becomes insulating due to the adsorbed inhibitor film^57^.

#### Atomic Force Microscopy (AFM)

**Figure 10:**
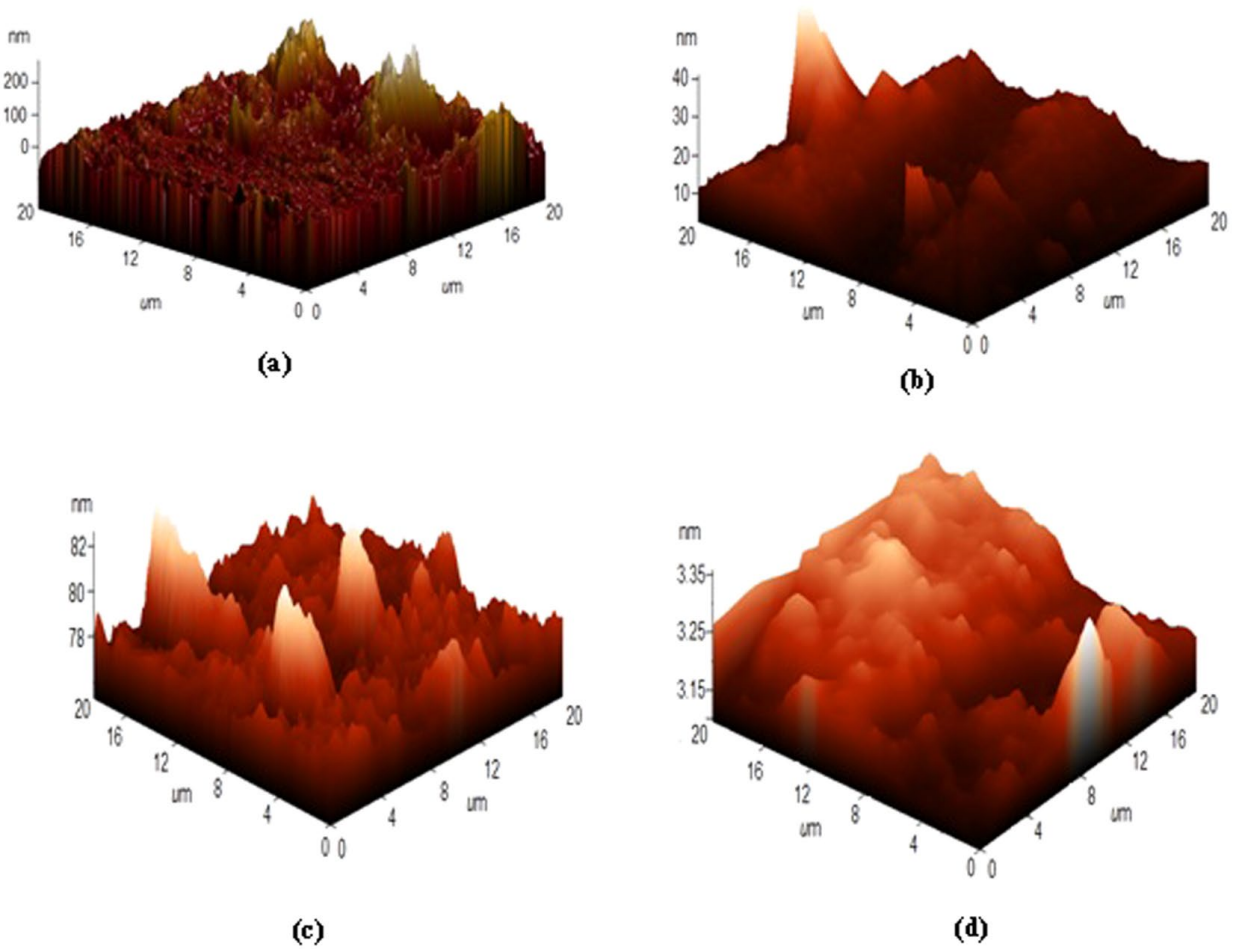
Atomic force microscopy images for (**a**) Blank-J55 steel (**b**) Blank-N80 steel (**c**) PM + J55 steel and (**d**) PM + N80 steel.

The 3-D AFM images of steel surfaces in the absence and presence of inhibitor are shown in Fig. 10a–d. In the absence of the inhibitor, the metal surface was damaged. The damage is strongly attributed to the dissolution of the oxide film and the maximum height scale reaching up to 200 nm and 82 nm for N80 and J55 steels respectively (Fig. 10a,b). However, in the presence of the inhibitor, the metal surface appears flatter, homogeneous and uniform, and the maximum height scale decreases to 40 nm and 3.35 nm for N80 and J55 steels, respectively (Fig. 10b,d)^58^. These results further support the formation of an inhibitor film over the metal surface.

### Quantum chemical calculations

**Figure 11:**
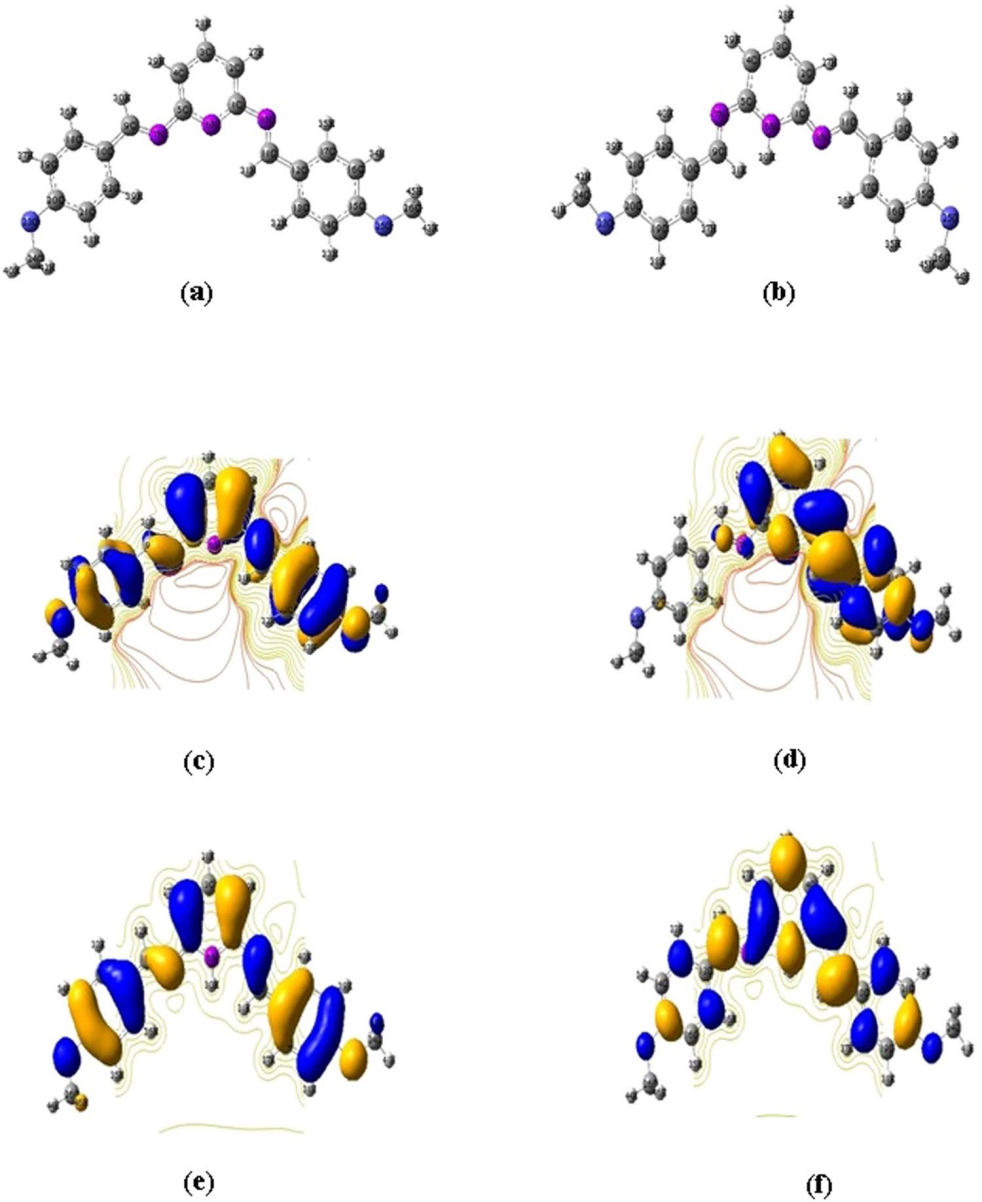
(**a**,**b**) Optimized geometries (**a**) neutral (**b**) protonated. (**c**,**d**) Frontier molecular orbitals of the neutral inhibitor (**c**) HOMO (**d**) LUMO. (**e**,**f**) Frontier molecular orbitals of the protonated inhibitor (**c**) HOMO (**d**) LUMO.

**Figure 12:**
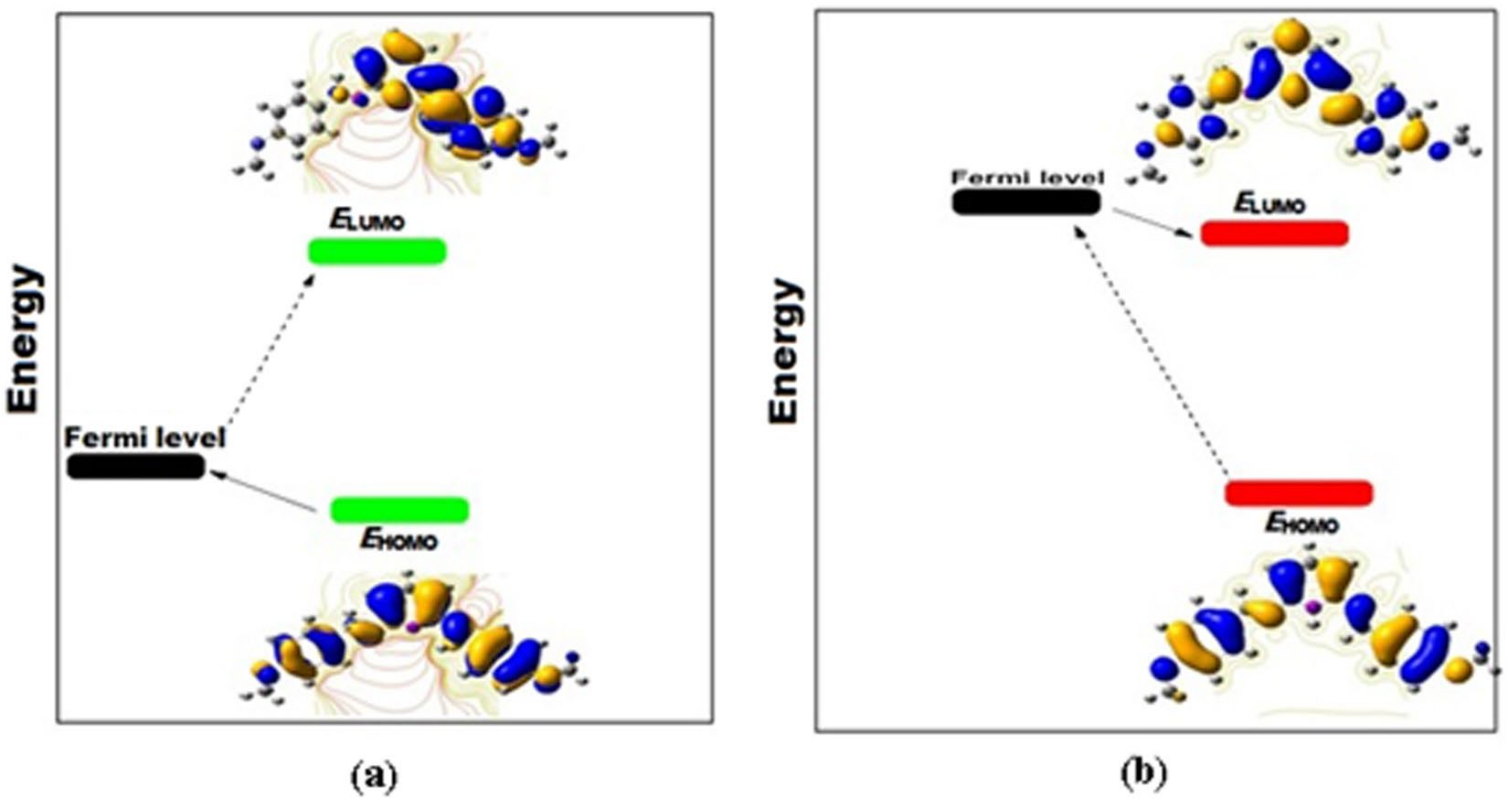
(**a**,**b**) Frontier orbital energetic positions of inhibitor molecule with iron surface (**a**) neutral inhibitor (**b**) protonated inhibitor.

The optimized geometry and frontier orbital energy of neutral and protonated inhibitor are shown in Fig. 11a–f. The quantum chemical parameters are tabulated in Table 4. The adsorption of inhibitor molecules over the metal surface depends upon the position of the frontier orbital energy level between the inhibitor molecules and the Fermi level of the iron metal^59^.The frontier orbital energies of inhibitor molecules in neutral and protonated forms and the Fermi level of iron are shown in Fig. 12a,b. Figure 12a shows that in the neutral form of the inhibitor, the *E*_HOMO_ energy level is at −5.440 eV, very close to the Fermi level of iron, i.e., −5.177 eV. However, the *E*_LUMO_ energy level is at −1.577 eV, far away from the Fermi level of iron. Therefore, the transfer of an electron from the HOMO energy level to the iron surface can easily take place. The energy gap between the Fermi level of iron and the *E*_LUMO_ of the inhibitor molecule is large. Thus, the transfer of an electron from the iron surface to the LUMO orbital of the inhibitor molecule is very difficult.

**Table 4:** Calculated quantum chemical parameters of the inhibitor.

Inhibitors	*E*_HOMO_	*E*_LUMO_	*χ*	*η*	*∆N*
PM	−5.440	−1.577	3.508	1.931	0.339
PM^+^	−8.540	−5.616	7.078	1.462	−0.772

All energy values are in eV; ^b^σ is in eV^−1^; PM^+^ is the protonated inhibitor.

In the case of the protonated form of the inhibitor molecule (Fig. 12b), the Fermi level of iron (−5.177 eV) is very close to the *E*_LUMO_ energy level (−5.616 eV), while the *E*_HOMO_ energy level (−8.540 eV) is far from the Fermi level of iron. Thus, it could be very difficult for the electron transfer from the HOMO orbital to the iron surface to occur. However, the electron transfer occurs from the iron surface to the LUMO energy level. Furthermore, Table 4 reveals that the calculated Δ*N* value in the neutral form is positive, suggesting that the electron-donating capacity of the inhibitor molecules, while in the protonated form, becomes negative, which indicate that inhibitor molecules cannot donate electrons rather than accepting electrons from the metal.

### Fukui index analysis

Fukui index analysis was used to analyse the sites present over the inhibitor molecules, which are participating in the donor-acceptor interactions with the metal surface. The sites on the inhibitor molecules that donate and accept electrons are represented by *f*_k_^−^ (nucleophilic site) and *f*_k_^+^ (electrophilic site), respectively^60^. Thus, the higher the values of *f*_k_^−^ and *f*_k_^+^, the greater would be the electron donation and acceptance tendency, respectively. The calculated Fukui indices are presented in Table 5. In the studied inhibitor C(1), C(2), C(4), C(5), N(7), N (8), C(10), C(11), C(12), C(14), C(15), C(16), C(17), C(19), C(20), C(21), O(23) and O(25) atoms are more susceptible sites for donation of electron and C(1), C(2),C(3), C(4), N(6), N(8), C(11), C(12), C(13), C(15), C(17) and O(25), atoms are the most favourable sites for electron acceptance. Thus, heterocyclic rings along with the phenyl rings are most reactive sites for electron donor-acceptor interactions and facilitate inhibitor adsorption onto the steel surfaces.

**Table 5:** Calculated Fukui functions for the studied inhibitor molecules in neutral form.

Atoms	*f*_*k*_^*−*^	*f*_*k*_^+^
C1	0.0219	0.075
C2	0.1709	0.0115
C3	−0.2410	0.0423
C4	0.1735	0.0789
C5	0.0226	−0.0033
N6	−0.0039	0.0654
N7	0.0591	0.0003
N8	0.0824	0.1057
C9	0.0067	0.0005
C10	0.0480	0.0001
C11	0.0123	0.2949
C12	0.0860	0.0166
C13	0.0066	0.0681
C14	0.0452	−0.0067
C15	0.0523	0.1192
C16	0.0362	−0.0089
C17	0.0150	0.1240
C18	0.0037	0.0000
C19	0.0253	0.0000
C20	0.0293	0.0001
C21	0.0204	0.0000
C22	0.0085	0.0001
O23	0.0389	0.0000
C24	−0.0004	0.0000
O25	0.0693	0.0222
C26	−0.0006	−0.0001

### Molecular dynamics simulations

**Figure 13:**
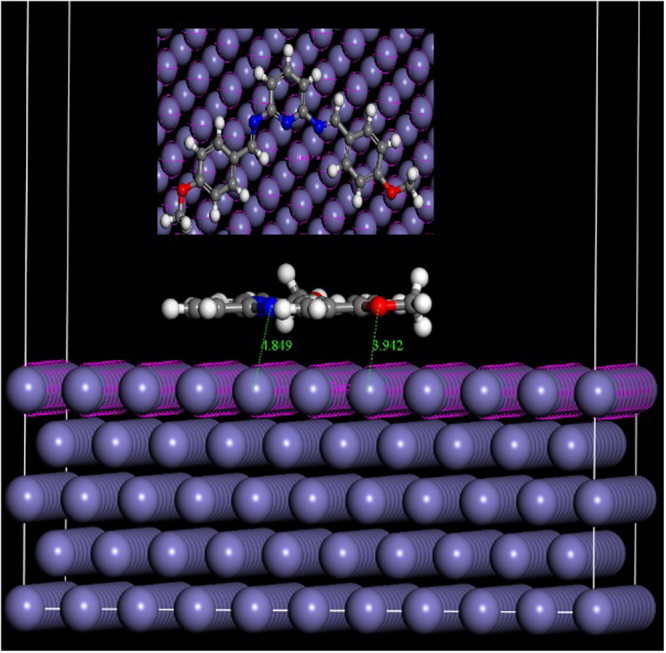
Top and side views of the most stable configurations for adsorption of inhibitor on Fe (110) surface calculated using Monte Carlo simulations.

The interaction between the metal and inhibitor was studied using molecular dynamics simulations, and the results are shown in Fig. 13. The parameters such as total energy, adsorption energy, rigid adsorption energy, and deformation energy are tabulated in Table 6. All energies are in kJ/mol.

**Table 6:** Adsorption energies for inhibitor on Fe (110) surface obtained using the molecular dynamic simulation (in kJ/mol).

System	Total Energy	Adsorption Energy	Rigid Adsorption Energy	Deformation Energy
Fe + PM	−30.208	−111.010	−8.716	−102.294

Inspection of the figure suggests that the inhibitor molecule adsorbs over the metal surface with a complete planar configuration. The adsorption energy in the present study is negative (−111.01 kJ/mol), which reveals stronger adsorption of the inhibitor molecule. Thus, the result of MD is in good agreement with the quantum chemical calculations and experimental results.

## Mechanism of corrosion mitigation

**Figure 14:**
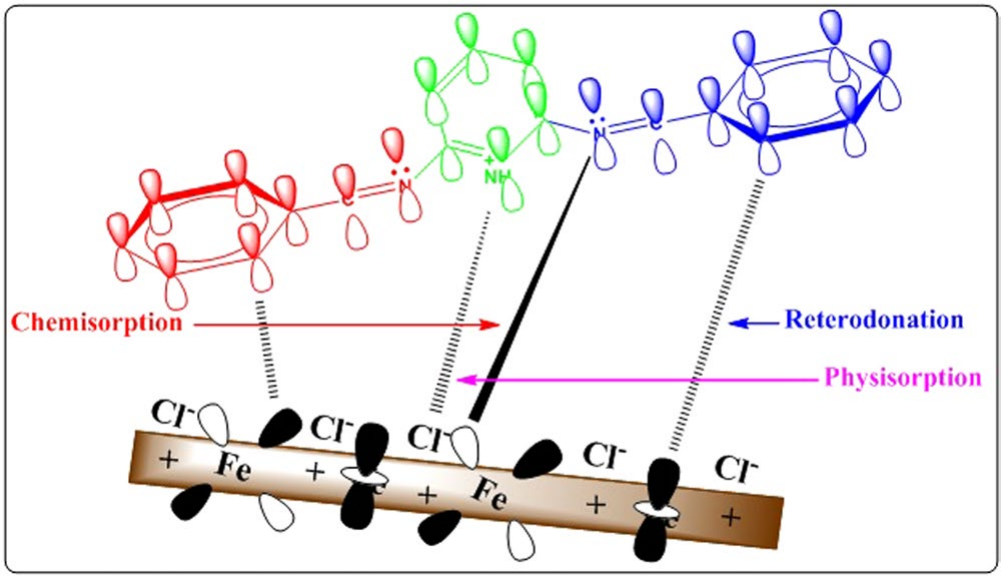
Mechanism of corrosion mitigation of steel in the presence of inhibitor in 3.5% NaCl solution saturated with CO_2_.

The adsorption of inhibitor molecule on the metal surface can be explained by the ideas obtained from the experimental in addition to quantum chemical study, and it could be taking place either physically or chemically or as a combination of both. Physical adsorption can be explained based on electrostatic interaction between the charged metal surface and the charged inhibitor molecules. Chemical adsorption occurs by donor-acceptor interactions between the lone pair electrons on the heteroatoms, π-electrons of multiple bonds as well as the phenyl group with the vacant d-orbitals of Fe^61,62^. Quantum chemical calculation shows that the inhibitor molecules exist in both neutral and protonated forms, so adsorption also occurs by a combination of both physical and chemical adsorption. In an acidic medium, the steel surface becomes positively charged after losing the electrons, as in Fig. 14. Thus, at the first stage, the Cl^−^ ions become adsorbed on the steel surface. Then protonated inhibitor molecules become adsorbed through electrostatic interactions (physical adsorption). At the same time, lone pair of electrons on the heteroatoms, and the π-electrons of the benzene ring are donated to vacant 3d-orbitals of iron atoms (chemical adsorption). Additionally, the filled metal orbitals give the electrons to the LUMO of the inhibitor molecules through reterodonation^63^.

## Conclusions

1. The R_p_ values increase with the increase in the concentration of the inhibitor, thus increasing inhibition efficiency.
2. Potentiodynamic polarization measurements indicate that the inhibitor action is mixed type.
3. The adsorption of the inhibitor on the J55/N80 steel surface obey the Langmuir adsorption isotherm.
4. Δ*G*°_ads_ results reveal that the adsorption of the inhibitors on the metal surface is spontaneous.
5. The AFM, SEM, XRD and contact angle analyses show that the inhibition of J55/N80 steel corrosion occurs due to the formation of an inhibitor film.
6. Quantum chemical study reveals that the neutral form of the inhibitor can donate the electrons to the metal, and the protonated form can accept electrons from the metal. Molecular dynamic simulation also corroborated the experimental results.

## Electronic supplementary material

NMR and IR spectra of the studied inhibitor
