# Supplementary material for: An impending inhibitor useful for the oil and gas production industry: Weight loss, electrochemical, surface and quantum chemical calculation
Source: Sci Rep. 2017 Nov 2;7:14904. doi: 10.1038/s41598-017-13877-0 (PMC5668278; doi:10.1038/s41598-017-13877-0)
Supplement: Supplementary file 1 — NMR and IR spectra of the studied inhibitor [file 41598_2017_13877_MOESM1_ESM.doc]

**An impending inhibitor useful for the oil and gas production industry: Weight loss, electrochemical, surface and quantum chemical calculation**

Ambrish Singh^a,b*^, K. R. Ansari^c^, Xihua Xu^a^, Zhipeng Sun^a^, Ashok Kumar^d^, and Yuanhua Lin^a,b**^

^a^ School of Materials Science and Engineering, Southwest Petroleum University, Chengdu-610500, Sichuan, China.

^b^ State Key Laboratory of Oil and Gas Reservoir Geology and Exploitation, Southwest Petroleum University, Chengdu, Sichuan 610500, China.

^c^ Department of Chemistry, Indian Institute of Technology, Banaras Hindu University, Varanasi-221005, U.P., India

^d^ Department of Chemistry and Biochemistry, Arizona State University, Tempe, Arizona, 85287-1604, United States of America.

***Corresponding author**

E-mail: [yhlin28@163.com](mailto:yhlin28@163.com); [vishisingh4uall@gmail.com](mailto:vishisingh4uall@gmail.com)

Ph.No.: +86-13908085550; +86-18384155035


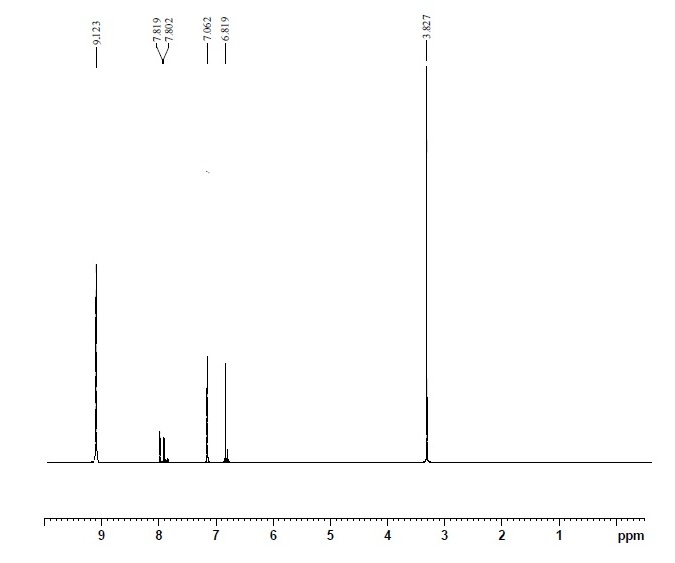


**Figure S1.** ^1^H NMR spectra


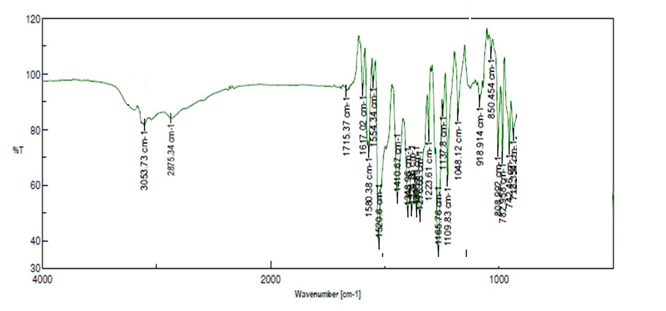


**Figure S2.** IR spectra
